# Methodological perspectives on the study of the health effects of unemployment – reviewing the mode of unemployment, the statistical analysis method and the role of confounding factors

**DOI:** 10.1186/s12874-022-01670-1

**Published:** 2022-07-21

**Authors:** Fredrik Norström, Anne Hammarström

**Affiliations:** 1 Department of Epidemiology and Global Health, Umeå University, 901 87 Umeå, Sweden; 2 Institute of Environmental Medicine, Occupational Medicine, Karolinska Institutet, Stockholm, Sweden

**Keywords:** Unemployment, Labour market, Propensity score weighting, G-computation, Biased estimates

## Abstract

**Introduction:**

Studying the relationship between unemployment and health raises many methodological challenges. In the current study, the aim was to evaluate the sensitivity of estimates based on different ways of measuring unemployment and the choice of statistical model.

**Methods:**

The Northern Swedish cohort was used, and two follow-up surveys thereof from 1995 and 2007, as well as register data about unemployment. Self-reported current unemployment, self-reported accumulated unemployment and register-based accumulated unemployment were used to measure unemployment and its effect on self-reported health was evaluated. Analyses were conducted with G-computation, logistic regression and three estimators for the inverse probability weighting propensity scores, and 11 potentially confounding variables were part of the analyses. Results were presented with absolute differences in the proportion with poor self-reported health between unemployed and employed individuals, except when logistic regression was used alone.

**Results:**

Of the initial 1083 pupils in the cohort, our analyses vary between 488–693 individuals defined as employed and 61–214 individuals defined as unemployed. In the analyses, the deviation was large between the unemployment measures, with a difference of at least 2.5% in effect size when unemployed was compared with employed for the self-reported and register-based unemployment modes. The choice of statistical method only had a small influence on effect estimates and the deviation was in most cases lower than 1%. When models were compared based on the choice of potential confounders in the analytical model, the deviations were rarely above 0.6% when comparing models with 4 and 11 potential confounders. Our variable for health selection was the only one that strongly affected estimates when it was not part of the statistical model.

**Conclusions:**

How unemployment is measured is highly important when the relationship between unemployment and health is estimated. However, misspecifications of the statistical model or choice of analytical method might not matter much for estimates except for the inclusion of a variable measuring health status before becoming unemployed. Our results can guide researchers when analysing similar research questions. Model diagnostics is commonly lacking in publications, but they remain very important for validation of analyses.

**Supplementary Information:**

The online version contains supplementary material available at 10.1186/s12874-022-01670-1.

## Introduction

Several studies have shown that unemployment is linked to deteriorated health [1, 2]. For studies on the health effect of unemployment, several methodological issues need to be handled and there are no previous study that have made a deeper investigation of this. In a review of the field, with the focus on self-assessed health, it was shown that many different statistical analysis methods, with a variety of explanatory variables, and study designs have been used for such studies [1].

In this study, we aim to investigate the key components of the analyses, including how unemployment is measured, by responding to slightly different research questions, the statistical analysis method and the variables that are included in analyses to improve estimates. In this study, we use current self-reported unemployment, and recent unemployment, both with self-reported and register-based information, as modes of unemployment. Current unemployment differs from the other modes in that it does not take accumulated exposure into account, while self-reported and register-based information can differ in many ways. Register-based unemployment is often seen as more objectively measured, while self-reported unemployment can be seen as more validly measured. Furthermore, self-reported data might not include shorter periods of unemployment between jobs, which is reported in register data. On the other hand, register data only include the number of days that people are registered as unemployed, and therefore lack information about unemployment when people are not entitled to unemployment benefits.

In a previous methodological review article of publications relating to unemployment and health, it was concluded that there were major weaknesses in many reporting aspects [3]. It was also reported that some of the reviewed manuscripts had probably used statistical analysis methods poorly fitted to the research question. A poor specification of the statistical analysis model could lead to biased results and consequently wrong conclusions [4]. A challenge in the specification is to identify a model that well describes the relationship between independent variables and outcome. Furthermore, it is essential to verify that independent variables are improving estimates of the effect of the main exposure on the outcome variable. It is therefore extremely important to understand whether data are having a relationship between them that can be well described based on the chosen method. Hence the motivation to use a thorough evaluation in this study.

To achieve an estimate of the causal effect with a small bias in observational studies, the choice of study design, and the statistical analysis to use needs to be well thought through so that it fits to the research question. Furthermore, the measurement of main exposure(s) and outcome(s) are essential; however, other variables that can play an essential role in the analysis are also crucial. For additional variables to contribute to the model, they need to confound the relation between exposure and outcome, and they should be chosen ahead of the analyses. In this study, the choice of the statistical analysis model is evaluated, as well as how sensitive results are for the selection of additional variables in the analysis. The key confounding factor to handle when unemployment and health are studied is previous health, as the risk of becoming unemployed also depends on health, and the unemployed tend to already have a poorer health [5]. This is usually referred to as *health selection* to unemployment, a phenomenon that has for instance been discussed and explained by Naimi et al. [6].

Within the field of unemployment and health, logistic regression is the most common statistical method, but other regression techniques are also common [1]. Despite the popularity of logistic regression, it seems that awareness of its limitations is generally poor, which have been verified by many published reviews showing a poor use and reporting of the method [3, 7]. These mistakes are likely to produce highly biased estimates. Methods based on so-called propensity scores, introduced in 1983 by Rosenbaum and Rubin [8], and G-computation have scarcely been used within public health [9], including our field of interest [1, 10]. Propensity score methods and traditional regression modelling, such as G-computation, tend to yield similar results [11], but this has yet to be investigated within our field of interest. Simulation studies have revealed that the variable selection has a bearing on the result when using propensity scores [12–15], but for unemployment and health this have not previously been investigated.

Our main aim is to evaluate the sensitivity of estimates based on the mode of unemployment and the choice of statistical model when the relationship between unemployment and health is studied.

## Methods

### Study design and participants

In our study, the Northern Swedish cohort was used [16], and our analyses are carried out on the same data set as was used in a previous publication, which showed that unemployment has a long-term negative health effect for those unemployed during young adulthood [10].

The Northern Swedish cohort was initiated in 1981, with all 1083 pupils in the last year of compulsory school during this year in a middle-sized town in Northern Sweden invited to participate in a study. Repeated questionnaires have been completed, including a matrix with detailed questions about labour market history since the last follow-up [17], for the cohort in four follow-ups (in 1983, 1986, 1995, and 2007) with a very high attrition rate (at least 94.3% of those still alive) on all occasions [16]. In this study, the follow-up questionnaires of the cohort in 1995 and 2007, which were answered by 1001 (92%) of the original participants, were combined with unemployment information from the longitudinal integration database for health insurance and labour market studies (acronym LISA) at Statistics Sweden. In LISA [18], information about labour market activity are obtained from the Swedish Public Employment Service. The Regional Ethical Board in Umeå, Sweden has approved the Northern Swedish cohort study. Further information about the cohort is available elsewhere [16].

### Definition of health

In our study, self-rated health from the 2007 questionnaire was used as the outcome variable, using the question (translated to English): “How do you assess your general state of health?”. Responses “fairly good” and “poor” were defined as poor and “good” was defined as good. The same question in the 1995 questionnaire was used identically to define *previous health*.

### Definitions of exposures

A labour market status variable was created in three different ways, referred to in this study as modes of unemployment, i) self-reported long-term unemployment, ii) register-based long-term unemployment, and iii) self-reported current unemployment. For the first, at least half a year of self-reported unemployment during last three years (1993–1995) was required. For the second, at least 6 months’ unemployment during 1992–1994 as reported in the LISA register database was required. In LISA, the information is limited to accumulated number of days of unemployment per year, which explains the choice of years as reported unemployment days in 1995 might have appeared after the participants’ response. The first two modes respond to the research question “What is the association between high exposure to unemployment in 1995 and self-rated health in 2007?”, while the last corresponds to the research question “What is the association between current unemployment 1995 and self-rated health in 2007?”.

Current unemployment required a tick at unemployed for the question “What is your current employment situation?”. Employed was defined as being active in the labour market, without unemployment, which we defined as either having “Full-time employment”, “Part-time (20–39 h a week) employment” or “Labour market measure”, for at least 1½ years, during the last three years. For current unemployment mode, a tick at any of the alternatives to the previously mentioned question defined employment. Response alternatives not considered as being in the labour market were “University/high-school”, “Other education”, “Casual job (< 20 h a week)”, “Sick leave”, “On parental leave”, and “Other”.

For all unemployment measures, analyses were conducted with unemployment episodes being allowed or not during the follow-up period. In the latter analyses, which we refer to as “censor” in current study, we required at least 1½ years in the labour market and no unemployment during follow-up for participants to be part of analyses. Being in labour market was defined similarly for the follow-up period as for the baseline definition from the 1995 survey. Details about questions and response alternatives in the two surveys for labour market status between surveys are presented in additional file 1.

If the aim is to get an unbiased estimate of the health effect of current unemployment exposure in 1995, both approaches have their weaknesses, censoring might exclude participants with recurrent unemployment caused by unemployment exposure in 1995, and with no censoring results might be affected by new episodes of unemployment or by participants moving out of the labour market. We consider both our alternatives to be proxies for our research questions, i.e. they will have limitations in measuring future health consequences of unemployment at young adulthood due to events during the follow-up.

### Confounding variables

For all potentially confounding variables, the responses to the 1995 questionnaire, when respondents were around 30 years of age, were used. As potential confounders, we chose the variables that had been most frequently used in similar studies according to a review in 2014 of manuscripts studying self-assessed health and unemployment [1]. Our approach can help to understand what variables can be urged to be used in similar research questions without risking non-negligible biases.

Body mass index (BMI) was derived from self-reported weight and height, and calculated as weight/length^2^; those with a BMI ≥ 30 kg/cm^2^ were defined as obese and those with a BMI between 25 and 30 kg/cm^2^ as overweight, and used as exposed groups. Education was divided into three groups: at most secondary education, upper secondary school and university degree, with university studies used as reference group. Based on the nomenclature used by Statistics Sweden [19], occupation was defined with low-medium white-collar work as reference group, and high white-collar and blue-collar workers as exposure groups. For marital status, married (including “living with cohabitant/partner”) was used as reference group and single as exposed group. For cash margin, availability of 13,000 SEK (corresponding to 1276 euro on 24^th^ November 2021) within a week was used as reference group and compared with no availability. For smoking, “not a current smoker” was used as reference group, and compared with “smoking at most 10 cigarettes a day”, and “smoking more than 10 cigarettes a day”. For social support, the instruments availability of social integration (AVSI) and availability of attachment (AVAT) were used [20]. The cut-offs for their reference groups were 13 or lower for AVSI and 10 or higher for AVAT. An index was calculated for alcohol intake based on six questions, a cut-off below 140, corresponding to low alcohol intake, was used as reference value. More details about the variables are available in a previous publication [10].

### Statistics

We restricted ourselves to participants in the 1995 labour market, defined as being employed or unemployed, with complete data for all study variables. This meant that we were able to include 55% to 74% of the originally invited pupils in our analyses. For the analyses, estimates based on propensity scores, G-computation and logistic regression were used. Among the 1001 participants, there were 113 participants who lacked information about any of the variables besides labour market status.

A risk difference was estimated based on propensity scores, using counterfactual arguments [21], with the weighting estimators suggested by Lunceford and Davidian [22]. The propensity score is the conditional probability of being assigned to the exposure group based on baseline covariates, i.e. for our study how likely it is to be unemployed based on values for covariates. We estimated the propensity scores with logistic regression. The counterfactual outcome for the difference estimators estimate E[Y(1)]-E[Y(0)], where E[Y(1)] corresponds to the expected effect if all individuals are unemployed, and E[Y(0)] corresponds to the expected effect if all individuals are employed. Thus, the risk difference corresponds to the marginal effect of becoming unemployed, i.e. the increase in absolute terms of poor health that unemployment would lead to.

Of the estimators, the first is the straightforward inverse probability weighting (IPW) estimator, the second is usually referred to as the augmented estimator (AUG), and the third is a doubly-robust estimator (DR). The doubly-robust estimator does not only include a weighting on the propensity score, but also makes use of the regression model from which the propensity scores are derived.

The estimators were:

$${RD}_{IPW}=\frac{1}{n}\left[\sum\limits_{i=1}^{n}\left(\frac{Y_{i}{X}_{i}}{PS_{i}}-\frac{Y_{i}\left(1-{X}_{i}\right)}{1-{PS}_{i}}\right)\right],{RD}_{AUG}={\left(\sum\limits_{i=1}^{n}\frac{X_{i}}{PS_{i}}\right)}^{-1}\sum\limits_{i=1}^{n}\frac{Y_{i}{X}_{i}}{PS_i}-{\left(\sum\limits_{i=1}^{n}\frac{1-{X}_{i}}{1-{PS}_{i}}\right)}^{-1}{\sum\limits_{i=1}^{n}}_{i}\frac{Y\left(1-{X}_{i}\right)}{1-{PS}_{i}}$$

and

$${RD}_{DR}=\sum \limits_{i=1}^n\left(\frac{Y_i{X}_i-\left({X}_i-{PS}_i\right){m}_1\left({X}_i\right)}{PS_i}-\frac{Y_i\left(1-{X}_i\right)+\left({X}_i-{PS}_i\right){m}_0\left({X}_i\right)}{1-{PS}_i}\right)$$

where *Y* refers to the outcome (self-rated health), *X* to the exposure (employed/unemployed), *PS* to the estimates of the propensity score. *m*_*0*_ and *m*_*1*_ refer to the estimated predictive value for the logistic regression used to derive propensity scores when only those defined as employed (*m*_*0*_) respectively unemployed (*m*_*1*_) are used to estimate beta coefficients.

For G-computation, logistic regression was first performed with all variables in the statistical model, including labour market status. The counterfactual outcome for the risk difference was calculated as the difference in effect if all individuals were unemployed with all individuals being employed. The Bootstrap technique with replacement was used to derive 10,000 replicates for the IPW and G-computing estimators and to calculate the mean square error (MSE) [23, 24]. The 2.5% and 97.5% percentiles were used to calculate 95% confidence intervals. For the doubly-robust estimator, some Bootstrap replicates had too few unemployed, which caused singularity problems and that logistic regression was not possible to conduct. Fewer replicates were then used.

The procedure for our analyses was to use all potentially confounding variables with logistic regression first in a “full” model, and thereafter a “reduced” model was applied with the significant variables in the full model for the self-reported long-term unemployment labour market mode. For the self-reported long-term unemployment mode, for both censored and non-censored analyses, we added or removed each potentially confounding variable individually. We choose this approach to show how collinear variables influence the effect estimates for the main exposure and for the logistic regression analyses for all variables.

R Studio was used for all statistical analyses, and its GLM procedure, with confidence intervals estimated using the profile likelihood estimator, was used for logistic regression estimates [25]. Statistical significance was defined at the 5% level.

## Results

### General characteristics

As only 22 participants who were unemployed in 1995 had experienced no unemployment during follow-up, the current unemployment mode with censoring could not provide reliable estimates and results are therefore not presented. For the unemployment modes that we used, the unemployment rate ranged from 8 to 27% (Table 1), which are explained by the definition of unemployed in the modes. For other variables, only minor differences (at most 7% between extremes) in the distribution within the variables were observed between the unemployment modes.

**Table 1:** Characteristics for the study populations

	**Unemployment mode**									
	*Self-reported long-term unemployment*				*Register-based long-term unemployment*				*Current* *unemployment*	
	*No censor*^*a*^		*Censored*^*a*^		*No censor*		*Censored*		*No censor*	
	*n*	%	*n*	%	*n*	%	*n*	%	*n*	%
**Labor market status**										
Employed	627	78%	522	84%	585	73%	488	80%	693	92%
Unemployed	178	22%	98	16%	214	27%	122	20%	61	8%
**Self-rated health 2007**										
* Poor*	286	36%	202	33%	283	35%	197	32%	262	37%
* Good*	519	64%	418	67%	516	65%	413	68%	492	63%
**Self-rated health 1995**										
* Poor*	186	23%	125	20%	179	22%	118	19%	164	22%
* Good*	619	77%	495	80%	620	78%	492	81%	590	78%
**Education level**^**b**^										
* Secondary education*	309	38%	253	41%	309	39%	251	41%	292	39%
* Upper secondary education*	146	18%	112	18%	144	18%	110	18%	134	18%
* University*	350	43%	255	41%	346	43%	249	41%	328	44%
**Marital status**										
* Married*	591	73%	468	75%	581	73%	460	75%	543	72%
* Single*	214	27%	152	25%	218	27%	150	25%	211	28%
**Occupation**										
Blue-collar	340	42%	246	40%	334	42%	239	39%	318	42%
Low white-collar	138	17%	103	17%	140	18%	104	17%	124	16%
Medium–high white-collar	327	41%	271	44%	325	41%	267	44%	312	41%
**Gender**										
* Man*	376	47%	283	45%	367	46%	278	46%	331	44%
* Woman*	429	53%	338	55%	432	54%	332	54%	423	56%
**Availability of Social Integration (AVSI)**										
* Low*	296	37%	203	33%	291	36%	197	32%	272	36%
* High*	509	73%	417	67%	508	64%	413	68%	482	74%
**Availability of Attachment (AVAT)**										
* Low*	434	53%	321	52%	433	54%	315	52%	415	55%
* High*	371	47%	299	48%	366	46%	295	48%	339	45%
**Cash margin**										
* Access*	640	80%	514	83%	639	80%	508	83%	605	80%
* No access*	165	20%	106	17%	160	20%	102	17%	149	20%
**Smoking**										
* Not smoking*	561	70%	449	72%	558	70%	444	73%	528	70%
* Smoking* ≤ *10 cigarettes*	152	19%	113	18%	147	18%	108	18%	139	18%
* Smoking* > *10 cigarettes*	92	11%	58	9%	94	12%	58	10%	87	12%
**Alcohol intake**										
* Low*	399	50%	313	50%	389	49%	306	50%	360	48%
* High*	406	50%	307	50%	410	51%	304	50%	394	52%
**Body mass index**										
* Normal*	511	63%	393	63%	508	64%	387	63%	474	63%
* Overweight*	247	31%	191	31%	245	31%	188	31%	234	31%
* Obese*	47	6%	36	6%	46	6%	35	6%	46	6%

*Note*: Self-reported health is presented for both 1995 and 2007. Information for other during 1995

^a^ Censored individuals were during the follow-up period between autumn 1995 and autumn 2007 either unemployed or were active in the labor market for too short period

^b^ Secondary education corresponds to at most 2-years of secondary education, and upper-secondary education corresponds to 3–4 years of secondary education

### Estimates of the long-term effect of unemployment

For the unemployment modes, most estimates showed a statistically significant negative long-term effect of unemployment when it was compared with being employed (Table 2). The statistical models with all potential confounders had a higher point estimate than the models with only significant confounders, the only exceptions being the logistic regression and the G-computation estimator for the register-based unemployment mode with censoring. Thus, adding more variables mainly increased the estimated effect of unemployment. Applying censoring for unemployment episodes lowered the negative effect of unemployment for all analyses compared to not censoring for unemployment during follow-up, ranging from an absolute risk difference of 0.001 to 0.039 between these estimates.

**Table 2:** Long-term effect on self-rated health from unemployment at 40 years of age for different estimators and unemployment mode

**Unemployment** **mode**	**Unemployment during** **follow-up**^**b**^		**Estimator**^**a**^					
		*Employed / unemployed*	*Logistic regression*	*G-computation*	*IPW standard*	*IPW augmented*	*IPW* *doubly robust*	*MSE*^*c*^*,* *low/high*
*All confounders*^*d*^								
Self-reported long-term unemployment	No censor	627 / 178	1.90 (1.31–2.76)	0.134 (0.051–0.22)	0.129 (0.045–0.21)	0.128 (0.042–0.21)	0.125 (0.041–0.21)	0.0018/ 0.0019
	Censor	522 / 98	1.73 (1.07–2.81)	0.111 (0.008–0.22)	0.114 (0.001–0.25)	0.108 (-0.005–0.23)	0.124 (0.022–0.23)	0.0028/ 0.0038
Register-based long-term unemployment	No censor	585 / 214	1.65 (1.16–2.34)	0.103 (0.027–0.18)	0.104 (0.028–0.18)	0.102 (0.025–0.18)	0.099 (0.025–0.17)	0.0014/ 0.0015
	Censor	488 / 122	1.38 (0.88–2.17)	0.064 (-0.027–0.16)	0.067 (-0.025–0.17)	0.069 (-0.025–0.16)	0.074 (-0.015–0.17)	0.0022/ 0.0023
Current unemployment	No censor	693 /61	1.76 (0.97–3.18)	0.121 (-0.018–0.26)	0.199 (0.006–0.48)	0.253 (0.057–0.44)	0.252 (-0.003–0.43)	0.0052/ 0.0147
*Significant confounders*^*e*^								
Self-reported long-term unemployment	No censor	627 / 178	1.85 (1.28–2.67)	0.129 (0.049–0.21)	0.123 (0.042–0.20)	0.123 (0.041–0.20)	0.120 (0.040–0.20)	0.0018/ 0.0021
	Censor	522 / 98	1.74 (1.08–2.79)	0.113 (0.013–0.22)	0.103 (0.0005–0.21)	0.103 (0.0002–0.21)	0.101 (0.004–0.20)	0.0025/ 0.0027
Register-based long-term unemployment	No censor	585 / 214	1.63 (1.16–2.31)	0.102 (0.028–0.17)	0.096 (0.022–0.17)	0.097 (0.022–0.17)	0.095 (0.021–0.17)	0.0014/ 0.0014
	Censor	488 / 122	1.40 (0.89–2.16)	0.066 (-0.022–0.16)	0.059 (-0.029–0.15)	0.059 (-0.029–0.15)	0.057 (-0.028–0.15)	0.0020/ 0.0021
Current unemployment	No censor	693 /61	1.66 (0.94–2.91)	0.109 (-0.019–0.25)	0.156 (-0.003–0.33)	0.155 (-0.005–0.32)	0.163 (0.012–0.32)	0.0046 /0.0072

IPW = inverse probability weighting estimator; Risk differences are used for the G-computation and IPW estimators

The *p*-value is below 0.05 if 1 is not in the confidence interval for logistic regression estimator and 0 not in the confidence interval for other estimators. All variables but the outcome variable was measured in 1995

^a^ Confidence intervals, corresponding to the 2.5% and 97.5 Bootstrap percentiles, are presented within parentheses. Estimates represent the health effect on unemployed compared to employed individuals as represented with relative risk for logistic regression (above 0 a poorer health) and risk difference (above 1 a poorer health) for other estimators

^b^ Participants were censored or not censored if they experienced unemployment between 1995 and 2007

^c^ MSE = Mean squared error. The lowest and highest MSE for the estimators of the definition is presented

^d^ Analyzes are controlling for alcohol intake, Availability of Attachment (AVAT), Availability of Social Integration (AVSI), body mass index, cash margin, education level, gender, marital status, previous health status, occupation and smoking

^e^ Significant confounders were determined with logistic regression. Analyzes are controlling for education level, marital status, previous health status and occupation

For most unemployment modes, there was a rather small difference in effect estimates for the IPW estimators. The G-estimator gave a much smaller effect estimate for current unemployment mode than the IPW estimators while the difference was smaller for the other analyses, although in general greater than the one between the IPW estimators.

There were notable differences between estimates for the different unemployment definitions. The smallest effects were observed for the register-based long-term unemployment definition where the risk difference estimators ranged between 0.06–0.07 with censoring, while two IPW estimates of the current unemployment definition showed an effect as large as 0.25 poorer health for unemployed. For logistic regression, the estimates varied considerably with the odds ratio ranging from 1.4 to 1.9, with six of ten estimates giving statistical significance.

In additional file 2, we have presented characteristics for employed and unemployed. There were a different pattern for many of the background variables distribution for unemployment, which is to be expected considering the deviation in results presented.

### The influence of confounders on effect estimates

In Tables 3 and 4, results are shown based on variables included in the analysis. In them, model 1 presents the result when all potentially confounding variables are included in the analysis and model 13 presents a reduced model where only variables significant in the logistic regression analyses are included. Consequently, model 16 for instance will present the result when the statistical analysis method consists of all significant variables except previous health.

**Table 3:** Logistic regression estimates for self-reported long-term unemployment, with or without censoring for unemployment during follow-up

	Labor market status	Education level		Marital status	Prev. health	Occupation		Sex		Social network			Cash margin		Smoking			Alcohol		Body mass index	
**Model**	UE	Med	High	Single	Poor	Med	High	Male	AVAT high		AVSI low	No access		≤10		>10	High		Over-weight		Obese
**No censoring (n=805)**^**a**^																					
*Model 1 *	1.90*	0.57*	0.60*	1.65*	4.03*	0.75	0.54*	1.11	0.97		1.10	0.95		1.30		1.20	0.74		1.07		1.80
*Model 2*	1.83*	-	-	1.67*	3.93*	0.77	0.71	1.03	0.99		1.12	0.95		1.29		1.17	0.71		1.05		1.67
*Model 3*	1.92*	0.56*	0.59*	-	4.03*	0.75	0.54*	1.18	0.96		1.10	1.02		1.32		1.26	0.78		1.05		1.88
*Model 4*	1.93*	0.59*	0.66	1.65*	-	0.79	0.55*	1.08	0.90		1.02	1.15		1.51*		1.59	0.77		1.11		2.13*
*Model 5*	1.91*	0.65	0.84	1.66*	4.00*	-	-	1.13	0.98		1.10	1.08		1.34		1.28	0.71		1.06		1.72
*Model 6*	1.89*	0.58*	0.61*	1.67*	4.02*	0.73	0.54*	-	0.97		1.11	0.93		1.28		1.18	0.76		1.08		1.82
*Model 7*	1.90*	0.57*	0.60*	1.65*	4.04*	0.75	0.54*	1.11	-		1.10	0.95		1.30		1.20	0.74		1.07		1.80
*Model 8*	1.88*	0.57*	0.59*	1.65*	4.00*	0.74	0.54*	1.11	0.99		-	0.94		1.30		1.21	0.74		1.07		1.80
*Model 9*	1.89*	0.57*	0.60*	1.64*	4.01*	0.75	0.55*	1.11	0.97		1.11	-		1.30		1.20	0.74		1.07		1.80
*Model 10*	1.93*	0.56*	0.61*	1.67*	4.16*	0.74	0.53*	1.06	0.97		1.10	0.95		-		-	0.77		1.07		1.77
*Model 11*	1.86*	0.55*	0.58*	1.59*	3.99*	0.74	0.53*	0.99	0.98		1.09	0.95		1.23		1.15	-		1.06		1.84
*Model 12*	1.91*	0.58*	0.61*	1.68*	4.12*	0.74	0.55*	1.13	0.96		1.11	0.94		1.29		1.20	0.73		-		-
*Model 13*	1.85*	0.57*	0.54*	1.85*	4.13*	0.73	0.54*	-	-		-	-		-		-	-		-		-
*Model 14*	1.77*	-	-	1.61*	4.00*	0.77	0.70*	-	-		-	-		-		-	-		-		-
*Model 15*	1.89*	0.58*	0.61*	-	4.20*	0.72	0.53*	-	-		-	-		-		-	-		-		-
*Model 16*	2.00*	0.59*	0.70	1.69*	-	0.76	0.51*	-	-		-	-		-		-	-		-		-
*Model 17*	1.90*	0.65	0.87	1.65	4.20*	-	-	-	-		-	-		-		-	-		-		-
*Model 18*	1.85*	0.57*	0.60*	1.62*	4.14*	0.73	0.54*	1.01	-		-	-		-		-	-		-		-
*Model 19*	1.85*	0.57*	0.60*	1.62*	4.13*	0.73	0.54*	-	0.98		-	-		-		-	-		-		-
*Model 20*	1.87*	0.57*	0.61*	1.63*	4.17*	0.74	0.53*	-	-		1.09	-		-		-	-		-		-
*Model 21*	1.87*	0.57*	0.60*	1.63*	4.17*	0.72	0.53*	-	-		-	0.94		-		-	-		-		-
*Model 22*	1.83*	0.57*	0.60*	1.61*	4.02*	0.73	0.55*	-	-		-	-		1.21		1.15	-		-		-
*Model 23*	1.88*	0.59*	0.63*	1.70*	4.17*	0.73	0.55*	-	-		-	-		-		-	0.79		-		-
*Model 24*	1.86*	0.55*	0.59*	1.60*	4.05*	0.74	0.53*	-	-		-	-		-		-	-		1.07		1.82
*Crude *	2.06* (1.5-2.9)	0.79	1.12	1.76*	4.38*	0.77	0.57*	0.95	0.86		0.87	1.59		1.61*		1.89*	0.89		1.10		2.04*
**Censoring (n=620)**^**a**^																					
*Model 1 *	1.73*	0.60	0.50*	1.77*	4.54*	0.80	0.49*	1.10	1.05		1.14	0.92		1.50		0.85	0.79		1.11		1.80
*Model 2*	1.66*	-	-	1.77*	4.39*	0.88	0.73	1.02	1.08		1.15	0.91		1.46		0.84	0.75		1.09		1.71
*Model 3*	1.73*	0.60	0.51*	-	4.46*	0.81	0.50*	1.16	1.04		1.15	1.01		1.50		0.90	0.83		1.12		1.96
*Model 4*	1.71*	0.59	0.57*	1.71*	- -	0.88	0.49*	1.06	0.98		1.07	1.18		1.66		1.22	0.82		1.23		2.35
*Model 5*	1.72*	0.72	0.77	1.75*	4.55*	-	-	1.11	1.07		1.12	1.01		1.55		0.95	0.74		1.10		1.77
*Model 6*	1.72*	0.72	0.61*	1.79*	4.52*	0.78	0.49*	-	1.04		1.14	0.90		1.47		0.84	0.81		1.13		1.82
*Model 7*	1.74*	0.60	0.50*	1.77*	4.53*	0.80	0.49*	1.10	-		1.15	0.92		1.50		0.85	0.79		1.11		1.80
*Model 8*	1.72*	0.60	0.50*	1.78*	4.51*	0.79	0.49*	1.11	1.08		-	0.91		1.48		0.85	0.80		1.12		1.80
*Model 9*	1.73*	0.60	0.50*	1.76*	4.49*	0.81	0.49*	1.12	1.05		1.14	-		1.50		0.85	0.79		1.11		1.81
*Model 10*	1.76*	0.59	0.51*	1.75*	4.50*	0.81	0.49*	1.07	1.05		1.10	0.92		-		-	0.82		1.11		1.78
*Model 11*	1.70*	0.58	0.49*	1.74*	4.50*	0.79	0.47*	1.02	1.06		1.12	0.93		1.43		0.82	-		1.11		1.84
*Model 12*	1.77*	0.62	0.11*	1.82*	4.72*	0.78	0.49*	1.14	1.03		1.14	0.91		1.49		0.85	0.78		-		-
*Model 13*	1.77*	0.58*	0.62*	1.63*	4.27*	0.73	0.54*	-	-		-	-		-		-	-		-		-
*Model 14*	1.69*	-	-	1.61*	4.13*	0.77	0.70*	-	-		-	-		-		-	-		-		-
*Model 15*	1.82*	0.59*	0.62*	-	4.35*	0.71	0.53*	-	-		-	-		-		-	-		-		-
*Model 16*	1.88*	0.61*	0.72	1.71*	-	0.77	0.51*	-	-		-	-		-		-	-		-		-
*Model 17*	1.83*	0.66	0.88	1.65*	4.32*	-	-	-	-		-	-		-		-	-		-		-
*Model 18*	1.77*	0.58*	0.62*	1.62*	4.27*	0.73	0.54*	1.02	-		-	-		-		-	-		-		-
*Model 19*	1.77*	0.58*	0.62*	1.63*	4.27*	0.73	0.54*	-	1.00		-	-		-		-	-		-		-
*Model 20*	1.79*	0.58*	0.62*	1.63*	4.32*	0.74	0.54*	-	-		1.13	-		-		-	-		-		-
*Model 21*	1.77*	0.58*	0.62*	1.62*	4.26*	0.73	0.54*	-	-		-	1.02		-		-	-		-		-
*Model 22*	1.74*	0.59*	0.61*	1.60*	4.10*	0.73	0.55*	-	-		-	-		1.31		1.25	-		-		-
*Model 23*	1.81*	0.60*	0.64*	1.69*	4.31*	0.72	0.55*	-	-		-	-		-		-	0.81		-		-
*Model 24*	1.77*	0.56*	0.60*	1.60*	4.17*	0.74	0.53*	-	-		-	-		-		-	-		1.05		1.82
*Crude*	1.78* (1.1-2.8)	0.80	1.02	1.74*	4.54*	0.88	0.61*	0.94	1.02		0.97	1.45		1.69*		1.45	0.89		1.23		2.33*

^a^ Participants were censored (excluded) or not censored (included) if they experience unemployment between 1995 and 2007

UE=unemployed. Prev. health = previous health as measured in 1995. Estimates represent the health effect on unemployed (exposed) compared to employed (non-exposed) individuals

Models: Model 1 – controlling for alcohol intake, Availability of Attachment (AVAT), Availability of Social Integration (AVSI), body mass index, cash margin, education level, gender, marital status, previous health status, occupation and smoking; Model 2: model 1 - education level; Model 3: model 1 – marital status; Model 4: model 1 – previous health; Model 5: model 1 – occupation; Model 6: model 1 – sex; Model 7: model 1 – AVAT; Model 8: model 1 – AVSI; Model 9: model 1 - cash margin; Model 10: model 1 – smoking; Model 11: model 1 - alcohol intake; Model 12: model 1 – obesity; Model 13: significant terms in full model, i.e education level, marital status, previous health status and occupation; Model 14: model 13 - education level; Model 15: model 13 – marital status; Model 16: model 13 – previous health; Model 17: model 13 - occupation; Model 18: model 13 + sex; Model 19: model 13 + AVAT; Model 20: model 13 + AVSI; Model 21: model 13 + cash margin; Model 22: model 13 + smoking; Model 23: model 13 + alcohol intake; Model 24: model 13 + obesity. Note: “-“ means that this variable was excluded in analyses compared to either model 1 or 13 and “+” means that the variable was added to the significant variables in the analyses

**Table 4:** Estimates of the health effect of unemployment on health for different variable setups for the logistic regression, G-computation and inverse propensity weighting (IPW) estimators for self-reported long-term unemployment

Model	Unemployment during follow-up^a^	Risk difference			
		*G-computation*	*IPW standard*	*IPW augmented*	*IPW* *doubly robust*
*Model 1 (full)*^*b*^	No censor	0.134*	0.129*	0.128*	0.125*
	Censor	0.111*	0.114*	0.108	0.124*
*Model 2:* *model 1—education level*	No censor	0.126*	0.130*	0.130*	0.129*
	Censor	0.103*	0.117	0.115	0.126*
*Model 3:* *model 1 – marital status*	No censor	0.138*	0.136*	0.136*	0.133*
	Censor	0.111*	0.116	0.109	0.131*
*Model 4:* *model 1—previous health*	No censor	0.148*	0.145*	0.142*	0.143*
	Censor	0.117*	0.119*	0.113	0.125*
*Model 5:* *model 1—occupation*	No censor	0.136*	0.127*	0.128*	0.125*
	Censor	0.111*	0.116*	0.110*	0.120*
*Model 6:* *model 1—sex*	No censor	0.133*	0.135*	0.136*	0.129*
	Censor	0.109*	0.121*	0.122	0.122*
*Model 7:* *model 1 – AVAT*	No censor	0.134*	0.128*	0.127*	0.122*
	Censor	0.112*	0.105*	0.099*	0.107*
*Model 8:* *model 1 – AVSI*	No censor	0.132*	0.132*	0.129*	0.127*
	Censor	0.109*	0.110*	0.103	0.118*
*Model 9:* *model 1—cash margin*	No censor	0.133*	0.120*	0.118*	0.117*
	Censor	0.110*	0.104*	0.096	0.115*
*Model 10:* *model 1 – smoking*	No censor	0.137*	0.126*	0.125*	0.121*
	Censor	0.115*	0.115*	0.108*	0.120*
*Model 11:* *model 1—alcohol intake*	No censor	0.129*	0.125*	0.128*	0.125*
	Censor	0.107*	0.111*	0.111*	0.124*
*Model 12:* *model 1—obesity*	No censor	0.135*	0.132*	0.131*	0.130*
	Censor	0.115*	0.118	0.115	0.125*
*Model 13: significant terms in full model*^*c*^	No censor	0.129*	0.123*	0.123*	0.120*
	Censor	0.113*	0.103*	0.103*	0.101*
*Model 14: model 13—education level*	No censor	0.121*	0.127*	0.125*	0.126*
	Censor	0.105*	0.107*	0.107*	0.107*
*Model 15: model 13 – marital status*	No censor	0.136*	0.133*	0.133*	0.130*
	Censor	0.116*	0.107*	0.106*	0.105*
*Model 16: model 13 – previous health*	No censor	0.159*	0.154*	0.152*	0.153*
	Censor	0.132*	0.121*	0.121*	0.120*
*Model 17: model 13—occupation*	No censor	0.136*	0.123*	0.123*	0.122*
	Censor	0.116*	0.105*	0.104*	0.102*
*Model 18: model 13* + *sex*	No censor	0.130*	0.118*	0.117*	0.116*
	Censor	0.114*	0.094	0.093	0.096
*Model 19: model 13* + *AVAT*	No censor	0.129*	0.123*	0.122*	0.121*
	Censor	0.112*	0.107*	0.107*	0.107*
*Model 20: model 13* + *AVSI*	No censor	0.131*	0.122*	0.122*	0.118*
	Censor	0.115*	0.105*	0.105*	0.102*
*Model 21: model 13* + *cash margin*	No censor	0.131*	0.132*	0.132*	0.130*
	Censor	0.115*	0.115*	0.114*	0.111*
*Model 22: model 13* + *smoking*	No censor	0.126*	0.126*	0.128*	0.126*
	Censor	0.109*	0.106*	0.107*	0.106*
*Model 23: model 13* + *alcohol intake*	No censor	0.132*	0.127*	0.126*	0.122*
	Censor	0.115*	0.105*	0.105*	0.102*
*Model 24: model 13* + *obesity*	No censor	0.129*	0.122*	0.122*	0.118*
	Censor	0.110*	0.097	0.097	0.096

*Note*: “- “ means that this variable was excluded in analyses compared to either model 1 or 13 and “ + ” means that the variable was added to the significant variables in the analyses

^*^ p < 0.05 based on the 2.5% and 97.5 Bootstrap percentiles

^a^ Participants were censored (*n* = 620) or not censored (*n* = 805) for unemployment during the follow-up period between 1995 and 2007. ^b^ Full model controlling for alcohol intake, Availability of Attachment (AVAT), Availability of Social Integration (AVSI), body mass index, cash margin, education level, gender, marital status, previous health status, occupation and smoking.

^c^ The significant variables in model 13–24 were education level, marital status, previous health status and occupation. IPW = inverse probability weighting estimator. Estimates represent the health effect on unemployed compared to employed individuals where estimate above 0 is poorer health for unemployed

For the self-reported long-term unemployment definition with no censoring, there were statistically significant odds ratios for all 24 models, with some variation dependent on the included variables, with odds ratios ranging between 1.77 and 2.00 for unemployment compared with employment (Table 3). The highest odds ratios occurred when previous health was removed in model 16, but still not higher than the crude odds ratio (2.06). For the definition with censoring, the odds ratios ranged between 1.66 and 1.88 for the different models. The crude odds ratio (1.78) was within this range. Of odds ratios other than the one for labour market status, mainly occupation and education affected each other when one of them was removed. The odds ratios then increased, in some cases even shifting to non-significance. The exclusion of previous health affected the odds ratios of some variables, including 0.03–0.15 lower odds ratio for labour market status.

For the risk difference estimators, there was an absolute increase in poor health due to unemployment that ranged from 9 to 16%, most of which was statistically significant, depending on the model and estimator (Table 4). Removal or addition of variables had some effect on estimates, where the largest effects resulted mainly from the removal of previous health from the confounders. When models were alternated with non-significant terms in the full model, the largest deviation was 0.014 for the risk difference (model 6 for censoring during follow-up) in comparison with model 1 or model 13. The deviation between models was at most 0.005 under this situation for the G-computation estimator.

## Discussion

In our study, we have shown that the unemployment mode has a considerable effect on estimates of the health effect of unemployment. It was interesting to notice that the absolute difference between self-reported and register-based unemployment was so substantial, ranging from as much as 2.5% to 3.1% for the risk difference estimators when there was no censoring for unemployment episodes during the follow-up period, and even greater when censoring for unemployment episodes. The results were similar for the logistic regression estimator too.

On the other hand, our evaluation of the model specification, i.e. the variables included in the statistical analysis model, yielded only rather marginal deviations in most cases. The exceptions were mainly related to the doubly-robust estimator, which is known to be sensitive to small samples, where we were able to identify some notable differences between the full model with all its variables and the model with only significant potential confounders, and estimates based on the current unemployment mode. For other comparisons between the full and the reduced model, the difference was no greater than 1.1%, and in most cases it was at most 0.6%.

Also, in the models where we evaluated the contribution of each potential confounder to the estimates, by either adding or removing a variable to or from the full or the reduced model, the effect estimates were at most marginally affected, with the exception of the presence of previous health in the analyses. Thus, our study shows that even if a correctly specified model is advantageous, the risk of bias is likely to be rather limited for similar research questions such as those we have investigated. To avoid biases due to the model set-up, it is paramount to take health selection in unemployment into consideration.

It is interesting that the more popular logistic regression estimator seems to be more sensitive to poor model assumptions than the propensity scores and G-computation methods. The comparison of our risk difference estimators, which showed that they yield mainly similar results, is very much in line with previous comparisons between propensity score methods and conventional multivariable methods [26]. Thus, it seems that the choice of the statistical method is not the main challenge in achieving estimates with a low bias.

Even if our study indicates that the statistical model might only cause small biases, the importance of the choice of variables is undoubtedly very important. Interestingly, in a review from 2014, only 6 of the 41 reviewed articles discussed the choice of statistical method, and if any of these publications discussed how to measure unemployment it was at least very rare [3]. Thus, most researchers need to be more informative about their analysis and describe the potential limitations from their choice of statistical model.

Our interest was in the estimate for labour market status. Interestingly, when studying how other estimates were affected by the model set-up, we observed that education and occupation were in some cases not significant on their own, while they were significant in combination, while the estimate of labour market was not as sensitive to the presence of these variables in the analysis. This behaviour of the estimates for education and occupation is contrary to the expectations when a variable is added, as collinearity is expected to rather lead to neither of two estimates being statistically significant than to an estimate going from not being statistically significant to being statistically significant when one of the variables is added. This finding further highlights the importance of a well thought through variable selection in the main analyses. Nevertheless, the rationale for including variables in the statistical analysis needs to be improved as has been highlighted in previous research [3, 27].

The importance of defining a research question that is accurately measured to well respond to it was one of the key issues in our study. Whether unemployment is self-reported or taken from a register should respond to the same research question and yield similar results. In our study, the deviation between results for register-based and self-reported labour market was rather large. To some extent, this might be explained by the register data not being available until the day of the survey. We do however think that the main explanation for the deviation is that differences in how data are collected lead to slightly different research questions being answered.

In our study, we did not focus on how other variables were measured, but it is equally important that they have a high validity for their measurement. We only used self-rated health to measure health. Results for unemployment and health have varied based on the health measure [1]. Thus, it is a limitation that we have not tried our analyses on other health outcomes to evaluate whether our conclusions would be valid. Additionally, it would have been valuable to assess how a measurement error for the potential confounders affects estimates. Its potential to bias results has been highlighted in the literature [28].

The estimates for current unemployment differed between our estimators. Even with no censoring, there are few unemployed, which is likely to explain large deviations. Also notable was that with all potential confounders in the model, the IPW estimates both deviated much and showed a notably larger effect between them. We do not recommend to use the current unemployment mode, both because too few are likely to currently be unemployed, and because the group of unemployed risks to be too heterogeneous. For all our estimators it is a limitation that we had few unemployed. However, we still had sufficiently many unemployed to fulfil the criteria of at least 10 cases for the least occurring outcome divided with the number of exploratory variables when we used only significant confounders to determine the propensities. As results also showed small variations with all confounders we expect also these results to be stable despite this limitation. This criterion as well as others have been recommended for logistic regression by for instance Bagley et al. [7].

Neither the accumulated unemployment spell during recent years nor current unemployment might cover how recent or current unemployment affects health in the long term. They may both be too limited in that the accumulated unemployment relates too much to historical unemployment and current to a very short and negligible unemployment spell for the person in question. Thus, it is likely to be more complicated to know how unemployment affects health, and, hence, also the importance of including health status at the time of unemployment into the analysis. The big discrepancy between the estimates from the measures used to collect unemployment in our study highlights the importance of a well thought-through measure. Thus, the main message in our work is to gain a deep understanding of how unemployment data are collected. Based on our experience from previous research, we recommend accumulated self-reported unemployment measured with a detailed retrospective matrix.

We have made a thorough analysis of different aspects of the analysis of unemployment. A possible limitation of our study is that only one population has been analysed and we have not tried to verify that it is the most suitable population for this kind of analysis. Another possible limitation is that despite different research questions used, we have still had a somewhat limited focus of the consequences of long-term unemployment on health. Even so, we believe that our results can have a major contribution for the future understanding of the priorities needed not only for this research topic but also for other research topics.

We cannot confirm that our conclusions are valid also for other countries. The labour market varies much between countries in regard to for instance unemployment benefits, but we think that such issues should have little consequence on the relationship between explanatory variables. We think that the similarity between estimates for our statistical methods will hold also for other populations as this pattern has commonly been shown in comparative studies between for instance logistic regression and propensity score based methods [26]. Collinearity between labour market measure and other explanatory variables is a potential problem for our analyses. However, it was only for previous health that estimates were affected and this variable is not correlated with labour market status, so even if labour market status might be correlated with other variables it is not likely to affect estimates for other populations.

## Conclusions

The choice of how to measure unemployment has had a rather large impact on effect estimates and if unemployment is incorrectly measured, and thus responding to a different research question, our study shows that results can vary substantially. There was even a notable deviation between results for register-based and self-reported unemployment, which probably did not depend only on the register not being able to capture unemployment until the day of the survey. In most cases, the choice of statistical method and variables in the model only had a very small effect on effect estimates, and only previous health needed to be handled well in the statistical analysis model. It should be noted that how variables interplay might differ between contexts and, thus, variables that did not affect our effect estimates might play a key role for other settings. It is therefore still of high importance that researchers put emphasis on model diagnostics and carefully evaluate whether the assumptions made in their analyses hold true and, more importantly, that any assumptions made are carefully taken care of, and better discussed and motivated in publications.

## Supplementary Information

**Additional file 1. **Questions about labour market status between follow-ups of the Northern Swedish Cohort in the surveys in 1995 and 2007.

**Additional file 2. **Characteristics for the study populations divided into employment groups for the unemployment modes.

## Data Availability

The questionnaires that were used for this study are available at https://ki.se/en/imm/the-northern-swedish-cohort. The datasets generated and/or analyzed during the current study are not publicly available because the Swedish Data Protection Act (1998:204) does not permit sensitive data on humans (like in our interviews) to be freely shared. The datasets are available based on ethical permission from the Regional Ethical board in Umeå, Sweden, from one of the co-authors (Anne Hammarström).

## Abbreviations

AVAT: Availability of attachment instrument
AVSI: Availability of social integration instrument
BMI: Body mass index
IPW: Inverse probability weighting
LISA: the longitudinal integration database for health insurance and labour market studies
MSE: Mean square error
