# Supplementary material for: Methodological perspectives on the study of the health effects of unemployment – reviewing the mode of unemployment, the statistical analysis method and the role of confounding factors
Source: BMC Med Res Methodol. 2022 Jul 21;22:199. doi: 10.1186/s12874-022-01670-1 (PMC9306210; doi:10.1186/s12874-022-01670-1)
Supplement: Supplementary file 1 — Additional file 1. Questions about labour market status between follow-ups of the Northern Swedish Cohort in the surveys in 1995 and 2007. [file 12874_2022_1670_MOESM1_ESM.docx]

## Questions about labour market status between follow-ups of the Northern Swedish Cohort in the surveys in 1995 and 2007

### 1995 survey

The question about past labour market status was: “What have you done since 1986, i.e. the year you turned 21?”. For each half year, from autumn 1986 to autumn 1995 (with some events mentioned to help the responder to remember the year), there were these 10 options: “university/high-school”, “other education”, “full time work, 40 hours a week”, “part time work, 20-39 hours a week”, “odd jobs, less than 20 hours a week”, “unemployed”, “labour market measure”, “sick leave”, “parental leave” and “other, what?”. The respondent made a tick on one or more of these for each half year.

### 2007 survey

The question about past labour market status survey was: “During which periods have you had a permanent contract resp. various forms of fixed-term employment or have been out of the labour market since 1996? Answer with one or several crosses for each spring term (= autumn) from January to July and autumn term (= autumn) from August to December. There are some events to help you to remember resp. year.”. For each half year from autumn 1986 to autumn 1995, there were these 11 options: “permanent position”, “self-employed”, the temporary contracts “project”, “substitute”, “needs employee”, “seasonal employment”, “other time-limited employment”, as well as “unemployed”, “labour market measure” and “outside labour market (including studies, travel, etcetera)”. The respondent made a tick on one or more of these for each half year.
