# Supplementary material for: Methodological perspectives on the study of the health effects of unemployment – reviewing the mode of unemployment, the statistical analysis method and the role of confounding factors
Source: BMC Med Res Methodol. 2022 Jul 21;22:199. doi: 10.1186/s12874-022-01670-1 (PMC9306210; doi:10.1186/s12874-022-01670-1)
Supplement: Supplementary file 2 — Additional file 2. Characteristics for the study populations divided into employment groups for the unemployment modes. [file 12874_2022_1670_MOESM2_ESM.docx]

**Table 1.** Characteristics for the study populations divided into employment groups for the unemployment modes

|  | **Unemployment mode** | | | | | | | | | | | | | | | | | | | | | | | | | | | | | | | | | | | | | | | | | | | | | | |  |  |  |  |
| --- | --- | --- | --- | --- | --- | --- | --- | --- | --- | --- | --- | --- | --- | --- | --- | --- | --- | --- | --- | --- | --- | --- | --- | --- | --- | --- | --- | --- | --- | --- | --- | --- | --- | --- | --- | --- | --- | --- | --- | --- | --- | --- | --- | --- | --- | --- | --- | --- | --- | --- | --- |
|  | *Self-reported long-term unemployment* | | | | | | | | | | | | | | | | | | *Register-based long-term unemployment* | | | | | | | | | | | | | | | | | | | | | | *Current*  *unemployment* | | | | | | | | | | |
|  | *No censor^a^* | | | | | | | | | *Censored^a^* | | | | | | | | | *No censor* | | | | | | | | | | | | *Censored* | | | | | | | | | | *No censor* | | | | | | | | | |  |
|  | Emp | | | | UE | | | | | Emp | | | | | UE | | | | Emp | | | | | UE | | | | | | Emp | | | | | | UE | | | | Emp | | | | | UE | | | | |  |  |
| **Self-rated health 2007** | n | % | n | | | % | | n | | | % | | n | | | % | | n | | | % | | n | | | % | n | | | | | % | | n | | | % | | n | | | % | | n | | % | | |  |  |  |
| *Poor* | 199 | 32 | | 87 | | | 49 | | 159 | | | 30 | | 43 | | | 44 | | | 185 | | 32 | | | 98 | | | 46 | 149 | | | | 31 | | 48 | | | 39 | | | 231 | | 33 | | 12 | | 29 | | | | |
| *Good* | 428 | 68 | | 91 | | | 51 | | 363 | | | 70 | | 55 | | | 56 | | | 400 | | 68 | | | 116 | | | 54 | 339 | | | | 69 | | 74 | | | 61 | | | 462 | | 67 | | 30 | | 71 | | | | |
| **Self-rated health 1995** |  |  | |  | | |  | |  | | |  | |  | | |  | | |  | |  | | |  | | |  |  | | | |  | |  | | |  | | |  | |  | |  | |  | | | | |
| *Poor* | 130 | 21 | | 56 | | | 31 | | 100 | | | 19 | | 25 | | | 26 | | | 118 | | 20 | | | 61 | | | 29 | 90 | | | | 18 | | 48 | | | 39 | | | 143 | | 21 | | 21 | | 50 | | | | |
| *Good* | 497 | 79 | | 122 | | | 69 | | 422 | | | 81 | | 73 | | | 74 | | | 467 | | 80 | | | 153 | | | 71 | 398 | | | | 82 | | 94 | | | 77 | | | 550 | | 79 | | 40 | | 95 | | | | |
| **Education level^b^** |  |  | |  | | |  | |  | | |  | |  | | |  | | |  | |  | | |  | | |  |  | | | |  | |  | | |  | | |  | |  | |  | |  | | | | |
| *Secondary education* | 260 | 41 | | 49 | | | 28 | | 223 | | | 43 | | 30 | | | 31 | | | 242 | | 41 | | | 67 | | | 31 | 205 | | | | 42 | | 46 | | | 38 | | | 277 | | 40 | | 15 | | 36 | | | | |
| *Upper secondary education* | 104 | 17 | | 42 | | | 24 | | 90 | | | 17 | | 22 | | | 22 | | | 95 | | 16 | | | 49 | | | 23 | 82 | | | | 17 | | 28 | | | 23 | | | 122 | | 18 | | 12 | | 29 | | | | |
| *University* | 263 | 42 | | 87 | | | 49 | | 209 | | | 40 | | 46 | | | 47 | | | 248 | | 42 | | | 98 | | | 46 | 201 | | | | 41 | | 48 | | | 39 | | | 294 | | 42 | | 34 | | 81 | | | | |
| **Marital status** |  |  | |  | | |  | |  | | |  | |  | | |  | | |  | |  | | |  | | |  |  | | | |  | |  | | |  | | |  | |  | |  | |  | | | | |
| *Married* | 470 | 75 | | 121 | | | 68 | | 396 | | | 76 | | 72 | | | 73 | | | 443 | | 76 | | | 138 | | | 64 | 372 | | | | 76 | | 88 | | | 72 | | | 506 | | 73 | | 37 | | 88 | | | | |
| *Single* | 157 | 25 | | 57 | | | 32 | | 126 | | | 24 | | 26 | | | 27 | | | 142 | | 24 | | | 76 | | | 36 | 116 | | | | 24 | | 34 | | | 28 | | | 187 | | 27 | | 24 | | 57 | | | | |
| **Occupation** |  |  | |  | | |  | |  | | |  | |  | | |  | | |  | |  | | |  | | |  |  | | | |  | |  | | |  | | |  | |  | |  | |  | | | | |
| Blue-collar | 252 | 40 | | 88 | | | 49 | | 202 | | | 39 | | 44 | | | 45 | | | 235 | | 40 | | | 99 | | | 46 | 188 | | | | 39 | | 51 | | | 42 | | | 281 | | 41 | | 37 | | 88 | | | | |
| Low white-collar | 102 | 16 | | 36 | | | 20 | | 85 | | | 16 | | 18 | | | 18 | | | 100 | | 17 | | | 40 | | | 19 | 84 | | | | 17 | | 20 | | | 16 | | | 112 | | 16 | | 12 | | 29 | | | | |
| Medium–high white-collar | 273 | 44 | | 54 | | | 30 | | 235 | | | 45 | | 36 | | | 37 | | | 250 | | 43 | | | 75 | | | 35 | 216 | | | | 44 | | 51 | | | 42 | | | 300 | | 43 | | 12 | | 29 | | | | |
| **Gender** |  |  | |  | | |  | |  | | |  | |  | | |  | | |  | |  | | |  | | |  |  | | | |  | |  | | |  | | |  | |  | |  | |  | | | | |
| *Man* | 286 | 46 | | 90 | | | 51 | | 233 | | | 45 | | 49 | | | 50 | | | 276 | | 47 | | | 91 | | | 43 | 225 | | | | 46 | | 53 | | | 43 | | | 299 | | 43 | | 32 | | 76 | | | | |
| *Woman* | 341 | 54 | | 88 | | | 49 | | 289 | | | 55 | | 49 | | | 50 | | | 309 | | 53 | | | 123 | | | 57 | 263 | | | | 54 | | 69 | | | 57 | | | 394 | | 57 | | 29 | | 69 | | | | |
| **Availability of Social Integration (AVSI)** |  |  | |  | | |  | |  | | |  | |  | | |  | | |  | |  | | |  | | |  |  | | | |  | |  | | |  | | |  | |  | |  | |  | | | | |
| *Low* | 213 | 34 | | 83 | | | 47 | | 166 | | | 32 | | 37 | | | 38 | | | 201 | | 34 | | | 90 | | | 42 | 157 | | | | 32 | | 40 | | | 33 | | | 235 | | 34 | | 37 | | 88 | | | | |
| *High* | 414 | 66 | | 95 | | | 53 | | 356 | | | 68 | | 61 | | | 62 | | | 384 | | 66 | | | 124 | | | 58 | 331 | | | | 68 | | 82 | | | 67 | | | 458 | | 66 | | 24 | | 57 | | | | |
| **Availability of Attachment (AVAT)** |  |  | |  | | |  | |  | | |  | |  | | |  | | |  | |  | | |  | | |  |  | | | |  | |  | | |  | | |  | |  | |  | |  | | | | |
| *Low* | 333 | 53 | | 101 | | | 57 | | 274 | | | 52 | | 47 | | | 48 | | | 305 | | 52 | | | 128 | | | 60 | 253 | | | | 52 | | 62 | | | 51 | | | 377 | | 54 | | 38 | | 90 | | | | |
| *High* | 294 | 47 | | 77 | | | 43 | | 248 | | | 48 | | 51 | | | 52 | | | 280 | | 48 | | | 86 | | | 40 | 235 | | | | 48 | | 60 | | | 49 | | | 316 | | 46 | | 23 | | 55 | | | | |
| **Cash margin** |  |  | |  | | |  | |  | | |  | |  | | |  | | |  | |  | | |  | | |  |  | | | |  | |  | | |  | | |  | |  | |  | |  | | | | |
| *Access* | 520 | 83 | | 120 | | | 67 | | 441 | | | 84 | | 73 | | | 74 | | | 489 | | 84 | | | 150 | | | 70 | 414 | | | | 85 | | 94 | | | 77 | | | 580 | | 84 | | 25 | | 60 | | | | |
| *No access* | 107 | 17 | | 58 | | | 33 | | 81 | | | 16 | | 25 | | | 26 | | | 96 | | 16 | | | 64 | | | 30 | 74 | | | | 15 | | 28 | | | 23 | | | 113 | | 16 | | 36 | | 86 | | | | |
| **Smoking** |  |  | |  | | |  | |  | | |  | |  | | |  | | |  | |  | | |  | | |  |  | | | |  | |  | | |  | | |  | |  | |  | |  | | | | |
| *Not smoking* | 455 | 73 | | 106 | | | 60 | | 388 | | | 74 | | 61 | | | 62 | | | 423 | | 72 | | | 135 | | | 63 | 363 | | | | 74 | | 81 | | | 66 | | | 495 | | 71 | | 33 | | 79 | | | | |
| *Smoking ≤10 cigarettes* | 108 | 17 | | 44 | | | 25 | | 88 | | | 17 | | 25 | | | 26 | | | 101 | | 17 | | | 46 | | | 21 | 81 | | | | 17 | | 27 | | | 22 | | | 121 | | 17 | | 18 | | 43 | | | | |
| *Smoking >10 cigarettes* | 64 | 10 | | 28 | | | 16 | | 46 | | | 9 | | 12 | | | 12 | | | 61 | | 10 | | | 33 | | | 15 | 44 | | | | 9 | | 14 | | | 11 | | | 77 | | 11 | | 10 | | 24 | | | | |
| **Alcohol intake** |  |  | |  | | |  | |  | | |  | |  | | |  | | |  | |  | | |  | | |  |  | | | |  | |  | | |  | | |  | |  | |  | |  | | | | |
| *Low* | 322 | 51 | | 77 | | | 43 | | 270 | | | 52 | | 43 | | | 44 | | | 299 | | 51 | | | 90 | | | 42 | 253 | | | | 52 | | 53 | | | 43 | | | 331 | | 48 | | 29 | | 69 | | | | |
| *High* | 305 | 49 | | 101 | | | 57 | | 252 | | | 48 | | 55 | | | 56 | | | 286 | | 49 | | | 124 | | | 58 | 235 | | | | 48 | | 69 | | | 57 | | | 362 | | 52 | | 32 | | 76 | | | | |
| **Body mass index** |  |  | |  | | |  | |  | | |  | |  | | |  | | |  | |  | | |  | | |  |  | | | |  | |  | | |  | | |  | |  | |  | |  | | | | |
| *Normal* | 402 | 64 | | 109 | | | 61 | | 339 | | | 65 | | 54 | | | 55 | | | 378 | | 65 | | | 130 | | | 61 | 316 | | | | 65 | | 71 | | | 58 | | | 437 | | 63 | | 37 | | 88 | | | | |
| *Overweight* | 189 | 30 | | 58 | | | 33 | | 154 | | | 30 | | 37 | | | 38 | | | 175 | | 30 | | | 70 | | | 33 | 144 | | | | 30 | | 44 | | | 36 | | | 212 | | 31 | | 22 | | 52 | | | | |
| *Obese* | 36 | 6 | | 11 | | | 6 | | 29 | | | 6 | | 7 | | | 7 | | | 32 | | 5 | | | 14 | | | 7 | 28 | | | | 6 | | 7 | | | 6 | | | 44 | | 6 | | 2 | | 5 | | | | |

EMP = employed participants; UE = unemployed participants

^a^ Censored individuals were during the follow-up period between autumn 1995 and autumn 2007 either unemployed or were active in the labor market for too short period. ^b^ Secondary education corresponds to at most 2-years of secondary education, and upper-secondary education corresponds to 3–4 years of secondary education
Note: Self-reported health is presented for both 1995 and 2007. Information for other during 1995.
